# An interpretable machine learning model for predicting febrile seizures following enterovirus infection in children

**DOI:** 10.1080/07853890.2026.2696106

**Published:** 2026-07-10

**Authors:** Yonghan Luo, Yuemei Feng, Yan Guo, Yanchun Wang, Xueshan Xia, Yue Feng

**Affiliations:** ^a^ Faculty of Life Science and Technology, Kunming University of Science and Technology, Kunming, China; ^b^ Second Department of Infectious Diseases, Kunming Children’s Hospital, Kunming, Yunnan, China; ^c^ Department of Nutrition and Food Hygiene, School of Public Health, Kunming Medical University, Kunming, China; ^d^ Department of Reproductive Gynecology, NHC Key Laboratory of Healthy Birth and Birth Defect Prevention in Western China, First People’s Hospital of Yunnan Province, Kunming, Yunnan, China; ^e^ Yunnan Provincial Key Laboratory of Public Health and Biosafety, Kunming Medical University, Kunming, Yunnan, China

**Keywords:** Febrile seizures, Enterovirus, Machine learning, XGBoost, Pediatrics

## Abstract

**Objective:**

This study aims to develop an interpretable machine learning model for predicting the risk of FS in children with enterovirus (EV) infections and to implement it for clinical application.

**Methods:**

This retrospective study included 446 hospitalized children with EV infection (144 FS, 302 non‑FS). LASSO regression and BORUTA algorithm selected 15 key predictors from 53 clinical variables. Six models (logistic regression, KNN, Naive Bayes, MLP, random forest, XGBoost) were constructed and evaluated using AUC, sensitivity, specificity, F1 score, and decision curve analysis. SHAP values provided interpretability, and a Shiny web‑based calculator was developed.

**Results:**

The XGBoost model demonstrated the best predictive performance: the training set AUC reached 0.972 (95% CI: 0.958–0.987), with sensitivity of 0.892 and specificity of 0.905. The internal validation set achieved an AUC of 0.842 (95% CI: 0.757–0.926). DCA confirmed its strong clinical applicability. SHAP analysis identified key features contributing to the model: fever duration, disease course, immunoglobulin M, neutrophil count, fibrinogen, CD8+ T-cell percentage, aspartate aminotransferase, CD3+ T-cell percentage, procalcitonin, presence of hand-foot herpes lesions, CD19+ B-cell percentage, erythrocyte sedimentation rate, lymphocyte count, serum ferritin level, and alanine aminotransferase. The ‘Shiny’ calculator facilitates personalized risk assessment.

**Conclusion:**

The XGBoost predictive model developed in this study demonstrated both high accuracy and clinical interpretability. The associated web-based calculator offers a new tool for risk stratification and management of FS in children with EV infections.

## 1. Introduction

Febrile seizures (FS) are the most common convulsive disorder in childhood, typically occurring in children aged 6 months to 5 years, with an estimated incidence of 2–5% [1]. FS are defined as seizure episodes associated with fever (generally ≥38 °C) in the absence of intracranial infection or other identifiable causes[1,2]. Although most FS follow a benign and self-limiting course, their sudden onset can provoke significant anxiety in caregivers, often resulting in repeated emergency visits and hospitalizations [3]. Moreover, a subset of children experiencing severe FS may progress to epilepsy or other neurological disorders, posing long-term health risks[4,5]. Therefore, the early identification of children at high risk for FS is crucial for implementing targeted monitoring, enabling timely intervention, and alleviating the emotional and socioeconomic burden on families and healthcare systems.

Infections are the most common cause of FS, with most febrile episodes in affected children resulting from viral rather than severe bacterial infections [6–8]. Respiratory syncytial virus, rhinovirus, parainfluenza virus, influenza virus, coronavirus, adenovirus, and human herpesvirus have all been identified as potential etiological agents of FS [6,9,10]. Enterovirus (EV) infection is a frequent cause of febrile illnesses in children, and growing evidence in recent years highlights EV as an important and distinct trigger for FS [11,12]. The pathophysiological mechanisms by which EV induces FS are complex and multifactorial. Certain EV serotypes, such as EV-A71, exhibit direct neurotropism, while the virus-induced proinflammatory cytokine cascade may enhance neuronal excitability[13,14]. Importantly, EV-associated FS presents diagnostic challenges in distinguishing it from other serious central nervous system infections. Moreover, findings from this study suggest that EV-related FS may be associated with poorer clinical outcomes, as evidenced by the higher rate of Intensive Care Unit (ICU) admissions observed in the FS group. Therefore, early and accurate identification of children at high risk for FS following EV infection holds significant clinical value.

Machine learning (ML) models possess unparalleled advantages in handling multivariate data and complex interactions, enabling the generation of individualized risk scores. These capabilities have led to their widespread application in predicting clinical diseases and patient prognoses[15–17]. For instance, recent study has employed association rule mining to develop a prediction model for recurrent febrile seizures (RFS) in children, achieving 97% classification accuracy[18]. Similarly, another study utilized LASSO regression to build a predictive model for FS, achieving an AUC of 0.884 in both training and internal validation sets, demonstrating excellent predictive ability[19]. However, the complexity of ML algorithms often renders them “black boxes” with opaque decision-making processes. This lack of interpretability significantly hinders their acceptance and implementation in clinical practice[20,21]. To address this fundamental limitation and enhance both the clinical utility and transparency of ML models, SHapley Additive exPlanations(SHAP) values have been employed to elucidate the basis of model predictions[22]. Furthermore, a web-based Shiny application has been developed and deployed to operationalize the predictive model[23,24]. This tool translates a complex ML algorithm into a user-friendly and transparent clinical decision support system. By inputting readily available clinical parameters into the interface, clinicians can instantly obtain a quantified probability of FS occurrence and a clearly defined risk classification. This approach effectively transforms model outputs into actionable clinical insights, thereby overcoming the interpretability challenges of traditional black-box models.

Based on the considerations, this study used ML techniques to develop and rigorously validate a specialized predictive model for estimating the risk of FS in hospitalized children with confirmed EV infections. To bridge the gap between complex ML-based prediction and clinical practice, a user-friendly web-based calculator (Shiny App) was developed as a key translational tool. This model holds significant potential clinical value by improving risk stratification among children with EV infections, aiding in the reduction of unnecessary medical interventions, and alleviating caregiver anxiety.

## 2. Materials and methods

### 2.1. Study population

A total of 664 pediatric patients diagnosed with EV infection and admitted to Kunming Children’s Hospital between January 2019 and August 2024 were included in this study. This study was approved by the Ethics Review Committee of the Children’s Hospital of Kunming Medical University (Approval Number: 2025-03-316-k01). Due to the retrospective design of the study, the requirement for informed consent was waived by the ethics committee of Ethics Review Committee of the Children’s Hospital of Kunming Medical University. All methods were carried out in accordance with relevant guidelines and regulations, including the Declaration of Helsinki.

### 2.2. Inclusion and exclusion criteria

**Inclusion Criteria:**

Hospitalization with positive EV results confirmed by PCR in any one of the tested samples (throat swab, stool sample, or cerebrospinal fluid). The study was designed to include all pediatric patients from birth up to 18 years of age.

**Exclusion Criteria:**

1. Cases in which enteroviral infection was not the primary reason for hospitalization (n = 43)
2. Seizure cases with evidence of central nervous system infection based on cerebrospinal fluid or neuroimaging findings (n = 160)
3. Patients with a prior diagnosis of epilepsy or a history of FS (n = 15)
4. Patients with major comorbidities likely to confound seizure risk, including perinatal abnormalities, delayed psychomotor development, chromosomal abnormalities, congenital metabolic disorders, brain tumors, or history of intracranial surgery.
5. Cases with missing essential demographic or laboratory data.

In this study, the primary outcome was the occurrence of FS in children with EV infection. Patients who experienced FS were classified into the FS group, while those without FS were categorized into the non-FS group. The diagnosis of FS was based on the expert consensus on the diagnosis and management of FS[2]. For patients admitted to the pediatric ICU, the institutional criteria included: prolonged seizures lasting more than 15 min, recurrent seizures within 24 h, focal seizures accompanied by altered consciousness, or respiratory compromise requiring close monitoring.

### 2.3. Study variables and data extraction

Clinical data were extracted from the electronic medical records system of Kunming Children’s Hospital, including baseline characteristics (age, sex, weight, history of febrile seizures), type of infecting enterovirus (EV-A6, EV-A10, EV-A16, EV-A71, universal EV type), clinical symptoms (e.g. fever duration, disease course, rash distribution, gastrointestinal and respiratory symptoms, neurological signs), laboratory parameters (hematological, biochemical, inflammatory, and immunological markers as detailed in Table 1), and clinical outcomes (fever resolution time, length of hospital stay, ICU admission).

**Table 1.** Comparison of clinical characteristics between children with and without febrile seizures following enteroviral infection.

	Total (*n* = 446)	Non-febrile seizure (*n* = 302)	Febrile seizure (*n* = 144)	P
Baseline characteristics				
Age, Median (Q1, Q3), y	1 (1, 2)	1 (1, 2)	1 (1, 2)	0.801
Sex, n (%)				0.623
female	266 (60)	183 (61)	83 (58)	
male	180 (40)	119 (39)	61 (42)	
Weight, Median (Q1, Q3), kg	11 (9.8, 13)	11 (9.72, 13.45)	11 (10, 12.77)	0.965
History of previous febrile seizures				0.995
No	429 (96)	291 (96)	138 (96)	
Yes	17 (4)	11 (4)	6 (4)	
Type of infecting enterovirus				
EV-A6, n (%)				0.066
No	281 (63)	181 (60)	100 (69)	
Yes	165 (37)	121 (40)	44 (31)	
EV-A10, n (%)				0.113
No	403 (90)	278 (92)	125 (87)	
Yes	43 (10)	24 (8)	19 (13)	
EV-A16, n (%)				0.023
No	406 (91)	268 (89)	138 (96)	
Yes	40 (9)	34 (11)	6 (4)	
EV-A71, n (%)				0.019
No	435 (98)	291 (96)	144 (100)	
Yes	11 (2)	11 (4)	0 (0)	
EV-Universal type, n (%)				0.011
No	257 (58)	187 (62)	70 (49)	
Yes	189 (42)	115 (38)	74 (51)	
Symptoms				
Fever, n (%)				1
No	8 (2)	6 (2)	2 (1)	
Yes	438 (98)	296 (98)	142 (99)	
Fever duration, Median (Q1, Q3), d	2 (1, 4)	3 (2, 4)	1 (1, 2)	< 0.001
Disease course, Median (Q1, Q3), d	2 (1, 4)	3 (2, 5)	1 (1, 2)	< 0.001
Pharyngeal herpes lesions, n (%)				0.001
No	49 (11)	30 (10)	19 (13)	
Yes	397 (89)	272 (90)	125 (87)	
Hand-foot herpes lesions, n (%)				< 0.001
No	76 (17)	38 (13)	38 (26)	
Yes	370 (83)	264 (87)	106 (74)	
Buttocks and/or trunk herpes lesions, n (%)				0.138
No	365 (82)	241 (80)	124 (86)	
Yes	81 (18)	61 (20)	20 (14)	
Cough, n (%)				< 0.001
No	158 (35)	90 (30)	68 (47)	
Yes	288 (65)	212 (70)	76 (53)	
Sneezing/rhinorrhea, n (%)				0.654
No	368 (83)	247 (82)	121 (84)	
Yes	78 (17)	55 (18)	23 (16)	
Nasal obstruction, n (%)				0.963
No	410 (92)	277 (92)	133 (92)	
Yes	36 (8)	25 (8)	11 (8)	
Headache, n (%)				0.18
No	441 (99)	297 (98)	144 (100)	
Yes	5 (1)	5 (2)	0 (0)	
Vomiting, n (%)				< 0.001
No	366 (82)	233 (77)	133 (92)	
Yes	80 (18)	69 (23)	11 (8)	
Abdominal pain, n (%)				1
No	436 (98)	295 (98)	141 (98)	
Yes	10 (2)	7 (2)	3 (2)	
Inappetence, n (%)				0.184
No	412 (92)	275 (91)	137 (95)	
Yes	34 (8)	27 (9)	7 (5)	
Diarrhea, n (%)				1
No	423 (95)	286 (95)	137 (95)	
Yes	23 (5)	16 (5)	7 (5)	
Poor mental status, n (%)				0.315
No	408 (91)	273 (90)	135 (94)	
Yes	38 (9)	29 (10)	9 (6)	
Lethargy, n (%)				1
No	421 (94)	285 (94)	136 (94)	
Yes	25 (6)	17 (6)	8 (6)	
Irritability, n (%)				< 0.001
No	273 (61)	156 (52)	117 (81)	
Yes	173 (39)	146 (48)	27 (19)	
Shivering, n (%)				0.012
No	425 (95)	282 (93)	143 (99)	
Yes	21 (5)	20 (7)	1 (1)	
Neck rigidity, n (%)				0.053
No	342 (77)	223 (74)	119 (83)	
Yes	104 (23)	79 (26)	25 (17)	
Laboratory Tests				
WBC, Median (Q1, Q3), ×10^9^/L	9.81 (7.01, 13.32)	9.29 (6.12, 12.59)	11.13 (8.16, 14.01)	< 0.001
Hb, Median (Q1, Q3), g/L	128 (120, 134)	128 (120.25, 134)	126 (118.75, 131.25)	0.014
PLT, Median (Q1, Q3) ×10^9^/L	292 (239.25, 351.75)	295.5 (239.25, 355.25)	282 (239.75, 345.5)	0.499
Neutrophil count, Median (Q1, Q3) ×10^9^/L	5.48 (3, 9.11)	4.74 (2.68, 7.81)	7.14 (4.28, 10.34)	< 0.001
Lymphocyte count, Median (Q1, Q3) ×10^9^/L	2.78 (1.81, 3.89)	2.92 (1.92, 4.02)	2.36 (1.58, 3.56)	0.01
CRP, Median (Q1, Q3), mg/L	13.71 (3.95, 29.81)	15.1 (3.74, 32.86)	10.77 (4.29, 24.5)	0.14
PCT, Median (Q1, Q3), mg/L	0.32 (0.25, 0.67)	0.3 (0.25, 0.52)	0.42 (0.25, 1.12)	0.008
IL-6, Median (Q1, Q3),pg/L	4.78 (1.93, 8.34)	4.65 (1.95, 8.34)	5.57 (1.9, 8.34)	0.581
Ferritin, Median (Q1, Q3), ng/ml	114.08 (75.63, 127.47)	114.08 (79.4, 134.02)	100.75 (71.63, 114.65)	0.005
ESR, Median (Q1, Q3), mm/h	22.56 (15, 27)	22.56 (16, 29)	19 (14, 22.56)	< 0.001
IgA, Median (Q1, Q3), g/L	0.42 (0.27, 0.64)	0.42 (0.26, 0.71)	0.41 (0.29, 0.54)	0.4
IgG, Median (Q1, Q3), g/L	6.56 (5.33, 7.74)	6.51 (5.25, 7.83)	6.9 (5.6, 7.61)	0.514
IgM, Median (Q1, Q3), g/L	1.16 (0.92, 1.45)	1.18 (0.93, 1.52)	1.12 (0.9, 1.24)	0.004
ALT, Median (Q1, Q3), U/L	15 (12, 19)	15 (12, 19)	16 (13, 18.74)	0.037
AST, Median (Q1, Q3), U/L	36 (31, 42)	35 (30, 43)	38 (32.95, 42)	0.027
ALB, Median (Q1, Q3), g/L	42 (39.5, 43.98)	41.7 (39.23, 43.6)	43.35 (40.65, 44.42)	0.004
CD3+, Median (Q1, Q3), %	51.99 (45.19, 58.6)	51.99 (45.13, 60.72)	51.99 (45.43, 53.59)	0.04
CD3 + CD4+, Median (Q1, Q3), %	27.29 (21.54, 32.34)	27.24 (21.29, 33.11)	27.29 (22.3, 29.69)	0.745
CD3 + CD8+, Median (Q1, Q3), %	19.8 (15.3, 23.14)	19.8 (15.67, 23.83)	19.79 (14.98, 19.9)	0.01
CD19+, Median (Q1, Q3), %	29.56 (23.52, 34.24)	29.3 (22.37, 32.94)	29.56 (27.3, 36.37)	< 0.001
CD16 + 56+, Median (Q1, Q3), %	17.39 (11.54, 22.15)	17.39 (11.98, 23.34)	17.39 (10.62, 19.56)	0.203
CD4+/CD8+, Median (Q1, Q3), %	1.53 (1.05, 1.86)	1.46 (1.01, 1.81)	1.55 (1.12, 1.93)	0.072
TBil, Median (Q1,Q3), umol/L	7.9 (6, 9.8)	7.6 (5.8, 9.67)	8.61 (6.47, 10.03)	0.031
SCr, Median (Q1, Q3), umol/L	20.33 (16.25, 23.24)	20 (16, 24)	21.77 (17, 23)	0.168
CKMB, Median (Q1, Q3),U/L	21 (16.47, 25)	20 (16, 24)	21.72 (17.38, 26)	0.018
APTT, Median (Q1, Q3), s	35.99 (34.32, 39.38)	35.99 (34.62, 40.08)	35.99 (33.77, 38.3)	0.08
PT, Median (Q1, Q3), s	13.4 (12.4, 14.7)	13.3 (12.3, 14.7)	13.7 (12.8, 14.7)	0.083
FG, Median (Q1, Q3), g/L	3.79 (3.4, 4.16)	3.79 (3.5, 4.2)	3.79 (3.3, 4)	0.009
Outcomes				
Fever resolution time, Median (Q1,Q3), d	3 (2, 4)	3 (2, 5)	2 (1, 3)	< 0.001
Length of hospital stay, Median (Q1,Q3), d	6 (6, 7)	6 (6, 7)	6 (5, 7)	0.016
ICU admission, n (%)				0.034
No	429 (96)	295 (98)	134 (93)	
Yes	17 (4)	7 (2)	10 (7)	

*WBC (white blood cell count);Hb (hemoglobin);PLT (platelet count);CRP (C-reactive protein);PCT (procalcitonin);IL-6 (interleukin-6);ESR (erythrocyte sedimentation rate);IgA (immunoglobulin A);IgG (immunoglobulin G);IgM (immunoglobulin M);ALT (alanine aminotransferase);AST (aspartate aminotransferase);ALB (albumin);CD3+ (cluster of differentiation 3 positive);CD3 + CD4+ (cluster of differentiation 3 positive, cluster of differentiation 4 positive);CD3 + CD8+ (cluster of differentiation 3 positive, cluster of differentiation 8 positive);CD19+ (cluster of differentiation 19 positive);CD16 + 56+ (cluster of differentiation 16 positive, cluster of differentiation 56 positive).*

*CD4+/CD8+ (ratio of cluster of differentiation 4 positive to cluster of differentiation 8 positive);TBil (total bilirubin); SCr (serum creatinine); CKMB (creatine kinase-MB); APTT (activated partial thromboplastin time); PT (prothrombin time); FG (fibrinogen); ICU (Intensive Care Unit). Normal laboratory reference ranges for pediatric patients were as follows: WBC 4.0–12.0 × 10^9^/L, Hb 110–150 g/L, PLT 100–400 × 10^9^/L, CRP <10 mg/L, PCT <0.5 ng/mL, IL-6 < 7 pg/mL, ESR 0–20 mm/h, IgA 0.2–1.0 g/L, IgG 6.0–16.0 g/L, IgM 0.4–2.3 g/L, ALT <40 U/L, AST <40 U/L, ALB 35–50 g/L, CD3^+^ 50–70%, CD4^+^ 30–45%, CD8^+^ 20–35%, CD19^+^ 10–25%, CD16^+^56^+^ 5–20%, CD4/CD8 ratio 1.0–2.0, TBil <21 μmol/L, SCr 20–60 μmol/L, CKMB <25 U/L, APTT 28–42 s, PT 11–15 s, FG 2.0–4.0 g/L*.

### 2.4. Model development and validation

The study first assessed the predictive power of continuous variables using receiver operating characteristic (ROC) analysis. A heatmap was used to visualize correlations among variables, identifying strong positive or negative associations that might affect model construction. Subsequently, least absolute shrinkage and selection operator (LASSO) regression was employed to select variables with non-zero coefficients, representing those with strong predictive performance. The BORUTA algorithm was then applied to refine the variable set by identifying those with the highest importance.

Six machine learning models were developed based on the BORUTA-selected variables. Model performance was evaluated using metrics including area under the ROC curve (AUC), F1 score, accuracy, sensitivity, and specificity, and results were visualized using a heatmap. The optimal cut-off score (target sensitivity) for classification was determined by the Youden index derived from the ROC curve, which maximizes the sum of sensitivity and specificity. Decision Curve Analysis (DCA) was used to assess the clinical utility of each model. Finally, a web-based’Shiny’calculator was developed to predict the likelihood of FS associated with EV infection.

### 2.5. Statistical analysis

All data analyses were performed using R statistical software (version 4.4.1). Continuous variables with normal distribution were expressed as mean ± standard deviation (x ± s), and group comparisons were performed using independent samples t-tests. Non-normally distributed continuous variables were presented as median (interquartile range) [M (IQR)] and compared using the Mann–Whitney U test. Categorical variables were described as frequencies and percentages [n (%)], and intergroup differences were analyzed using the χ^2^ test or Fisher’s exact test. The level of statistical significance was set at α = 0.05.

## 3. Results

### 3. 1.Baseline characteristics

**Figure 1.**
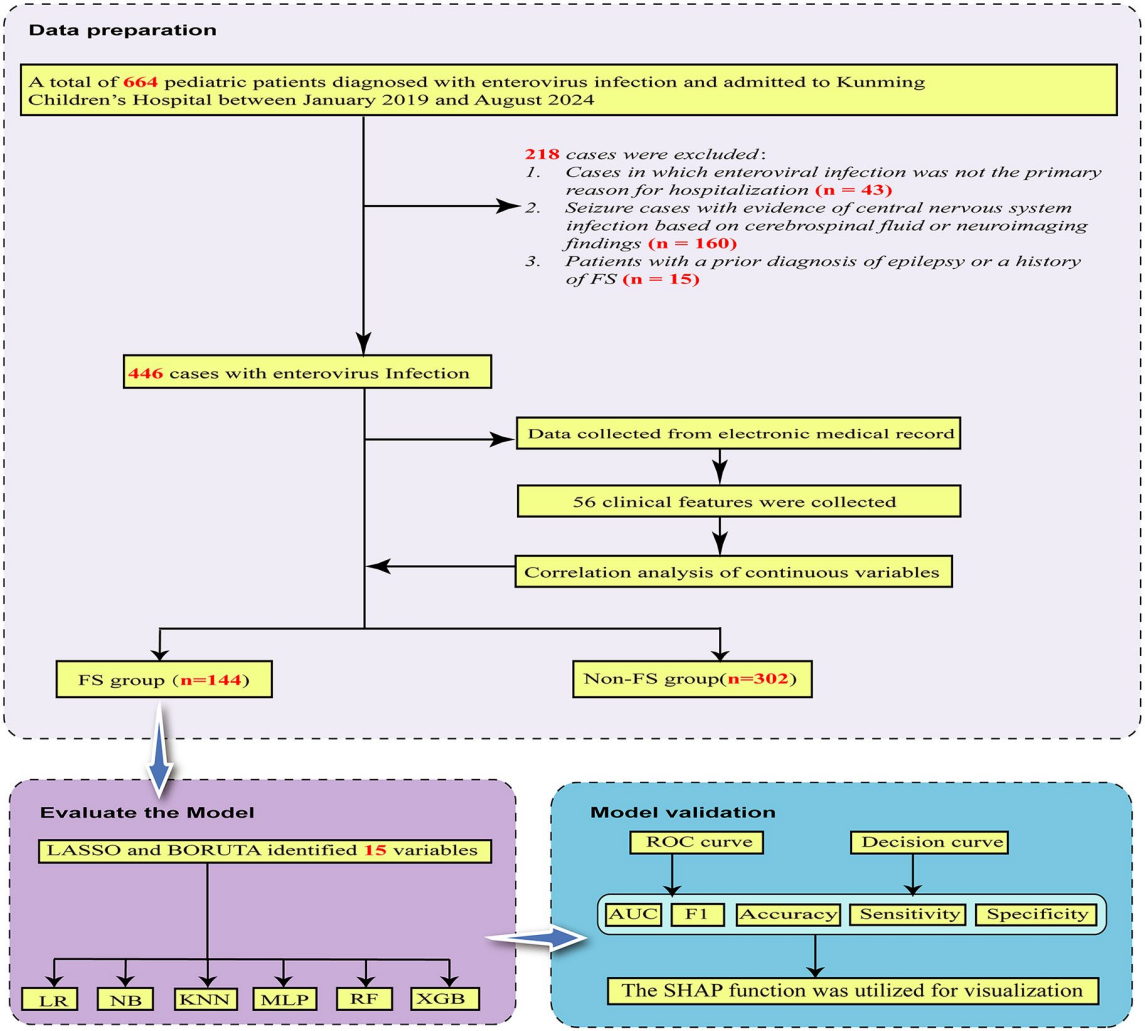
Study flowchart and model development process. Abbreviations: least absolute shrinkage and selection operator (LASSO) logistic regression (LR), naive Bayes (NB), k-nearest neighbors (KNN), multilayer perceptron (MLP), random forest (RF), and extreme gradient boosting (XGBoost, XGB), area under the receiver operating characteristic curve (AUC), decision curve analysis (DCA), Shapley additive explanations (SHAP).

A total of 664 cases were initially enrolled in this study. After applying exclusion criteria, 446 cases were ultimately included for analysis (the flowchart is shown in Figure 1). Among these, 302 cases (67.7%) were categorized as the non-FS group, while 144 cases (32.3%) comprised the FS group. Of the included patients, 266 (60%) were female and 180 (40%) were male, with a median age of 1 year. There were no statistically significant differences between the two groups in terms of sex distribution (*p* = 0.623) or age (*p* = 0.801). Similarly, no significant differences were observed regarding body weight and history of previous FS (*p* = 0.965 and *p* = 0.995, respectively).

**Figure 2.**
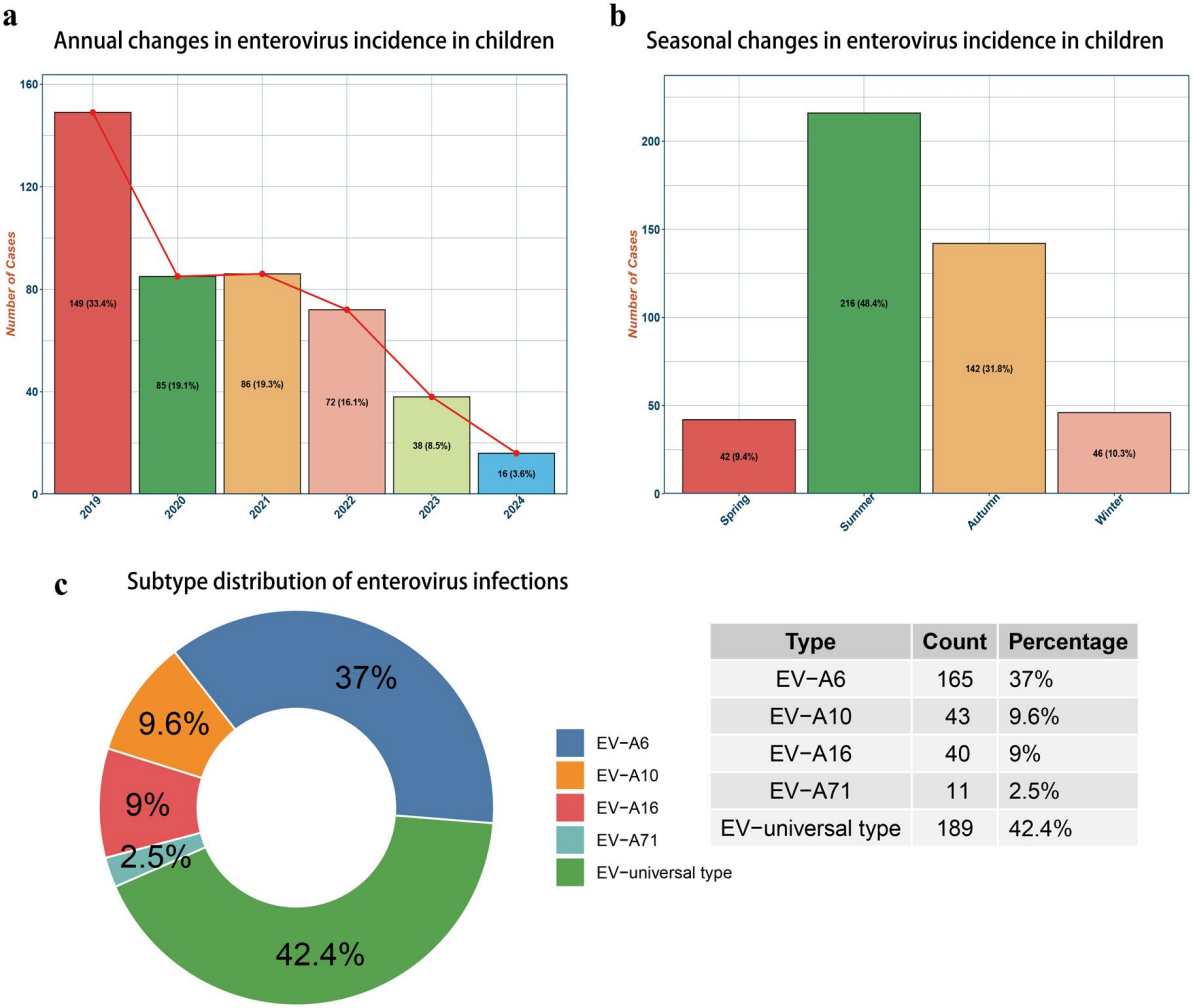
Incidence of Enterovirus Infection by Year **(a)** and Seasonal Distribution **(b)**. Subtype Distribution of Enterovirus Infections **(c)**.

Between 2019 and 2024, the number of pediatric cases of FS associated with EV infections demonstrated a consistent downward trend, declining from 149 cases (33.4%) in 2019 to just 16 cases (3.6%) in 2024 (Figure 2a). Seasonally, the highest incidence was observed in summer, accounting for 48.4% of all cases (Figure 2b). In terms of EV subtype distribution, EV- universal (42.4%) and EV-A6 (37%) were identified as the predominant strains, whereas subtypes such as EV-A10, EV-A16, and EV-A71 were less frequently detected (Figure 2c).

Regarding clinical symptoms, fever was observed in over 98% of patients in both groups. However, the duration of fever(pre-admission) and course of illness(pre-admission) was significantly shorter in the FS group compared to the non-FS group (*p* < 0.001 for both). Statistically significant intergroup differences were noted in the presence of pharyngeal herpes (*p* = 0.001), hand-foot herpes (*p* < 0.001), cough (*p* < 0.001), vomiting (*p* < 0.001), irritability (*p* < 0.001), and shivering (*p* = 0.012). No significant differences were observed in symptoms such as buttocks and/or trunk herpes lesions, sneezing/rhinorrhea, nasal obstruction, headache, abdominal pain, inappetence, diarrhea, poor mental status, lethargy, neck rigidity (all *p* > 0.05).

Laboratory investigations revealed statistically significant differences between the two groups in the following parameters: white blood cell count (WBC), neutrophil count, lymphocyte count, hemoglobin (Hb), procalcitonin (PCT), ferritin, erythrocyte sedimentation rate (ESR), immunoglobulin M (IgM), alanine aminotransferase (ALT), aspartate aminotransferase (AST), albumin (ALB), cluster of differentiation 3 positive (CD3^+^), cluster of differentiation 8 positive (CD8^+^), cluster of differentiation 19 positive (CD19^+^), total bilirubin (TBil), creatine kinase-MB (CKMB), fibrinogen (FG) (all *p* < 0.05). No significant differences were detected for C-reactive protein (CRP), interleukin-6 (IL-6), immunoglobulin A (IgA), immunoglobulin G (IgG), cluster of differentiation 4 positive (CD4^+^), CD4^+^/CD8^+^ ratio, serum creatinine (SCr), activated partial thromboplastin time (APTT), prothrombin time (PT) (all *p* > 0.05).

In terms of clinical outcomes, the fever resolution time was significantly shorter in the FS group (median 2 days vs. 3 days, *p* < 0.001), and hospitalization duration was also slightly reduced (*p* = 0.016). Notably, the ICU admission rate was significantly higher in the FS group compared to the non-FS group (7% vs. 2%, *p* = 0.034) **(Table 1).** Among FS patients admitted to the ICU, the main reasons were prolonged or recurrent seizures, and a small minority also presented with concurrent neurological complications.

### 3. 2. Selection of variables

**Figure 3.**
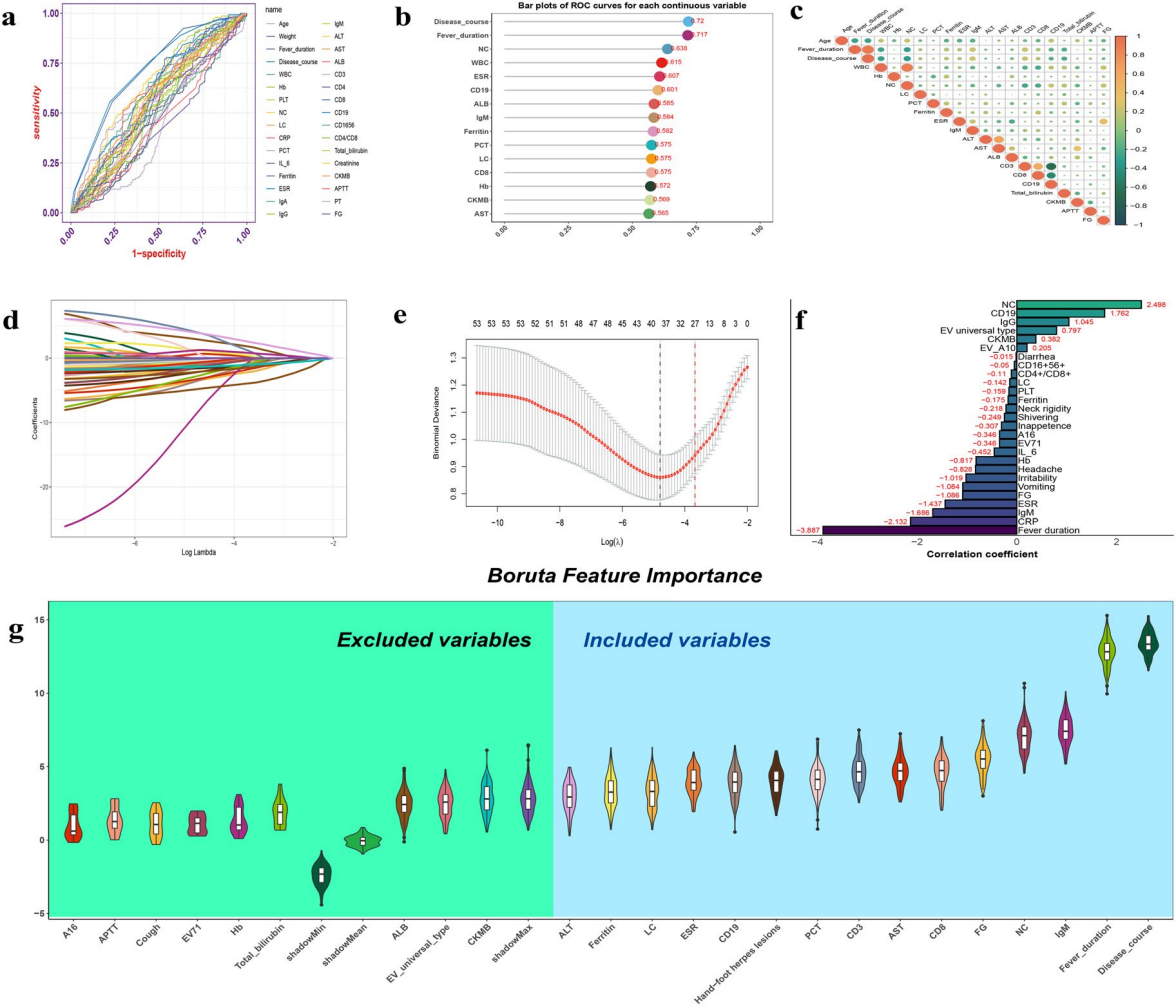
Variable Selection through LASSO Regression. **(a).** ROC Curve of a Single Continuous Variable for Enterovirus-Induced Febrile Seizures. **(b).** Bar Chart Ranking the Top Ten AUC Values for Enterovirus-Induced Febrile Seizures. **(c).** Heatmap Illustrating the Correlations Among Continuous Variables. Larger Pie Segments Indicate Stronger Correlations. **(d).** LASSO Coefficient Profiles of 53 Variables Plotted Against the Log(λ) Sequence. The Optimal Penalty Coefficient Lambda was Determined Using Tenfold Cross-Validation with a Minimization Criterion. **(e).** The Binomial Deviance Curve Plotted Against Log(λ), with Vertical Dotted Lines Indicating the Optimal Parameter (λ) for the LASSO Model. Twenty-seven Variables with Non-zero Coefficients were Selected Using the Lambda. Se Criterion. **(f).** Bar Chart Displaying the Coefficients for Variables with Non-zero Coefficients Selected by Lambda.Se. The Bars Represent the Strength of Association for Each Selected Variable, with Values Displayed at the End of Each Bar. **(g).** Variable Selection Using the ‘BORUTA’ Algorithm to Identify Key Variables Associated with the Prediction of Enterovirus-Induced Febrile Seizures. The Blue Area Represents Included Variables, and the Green Area Represents Excluded Variables.

 The initial set of variables comprised all variables (53 variables) listed in Table 1, excluding the outcome variables. Notably, only those variables available at or near the time of hospital admission were incorporated into the model. Variables such as total hospital length of stay and other post-admission metrics were deliberately excluded. To identify the most effective predictive variables, this study initially employed ROC curve analysis to assess the univariate predictive performance of all candidate variables **(**Figure 3A). A bar chart was used to visually present the top 15 variables ranked by AUC values. The results indicated that individual variables exhibited limited predictive power; notably, the highest-performing variable, “disease course,” achieved an AUC of only 0.72 **(**Figure 3B). This suggests that a multivariate combination approach is necessary to enhance model performance. In the correlation analysis of variables, the heatmap (Figure 3C) revealed significant correlations among certain variables (e.g. duration of fever and leukocyte count), indicating potential multicollinearity among the initial candidate features. To mitigate the impact of multicollinearity on model performance, the LASSO regression algorithm was applied to the 53 candidate variables for feature selection. Based on the lambda.se criterion, 27 variables with non-zero coefficients were ultimately retained **(Figures 3D–E),** and their relative weights were visualized through a coefficient plot (Figure 3F). However, clinical assessment revealed that these 27 LASSO-selected variables were of limited practical utility in predicting FS. Consequently, the study further employed the ‘BORUTA’ algorithm for feature selection, ultimately identifying 15 key predictors significantly associated with FS induced by EV infection (Figure 3G).

### 3. Model construction and validation

3.

 Based on the 15 key predictors identified by the BORUTA algorithm (Fever duration, Disease course, IgM [0.4–2.3 g/L], NC [1.5–8.0 × 10^9^/L], FG [2.0–4.0 g/L], CD8 [20–35%], AST [<40 U/L], CD3 [50–70%], PCT [<0.5 ng/mL], Hand foot herpes lesions, CD19 [10–25%], ESR [0–20 mm/h], LC [1.5–4.0 × 10^9^/L], Ferritin [30–400 ng/mL], ALT [<40 U/L]), we developed six distinct machine learning models using the training dataset: logistic regression, k-nearest neighbors (KNN), naïve Bayes, multilayer perceptron (MLP), random forest, and XGBoost.

**Figure 4.**
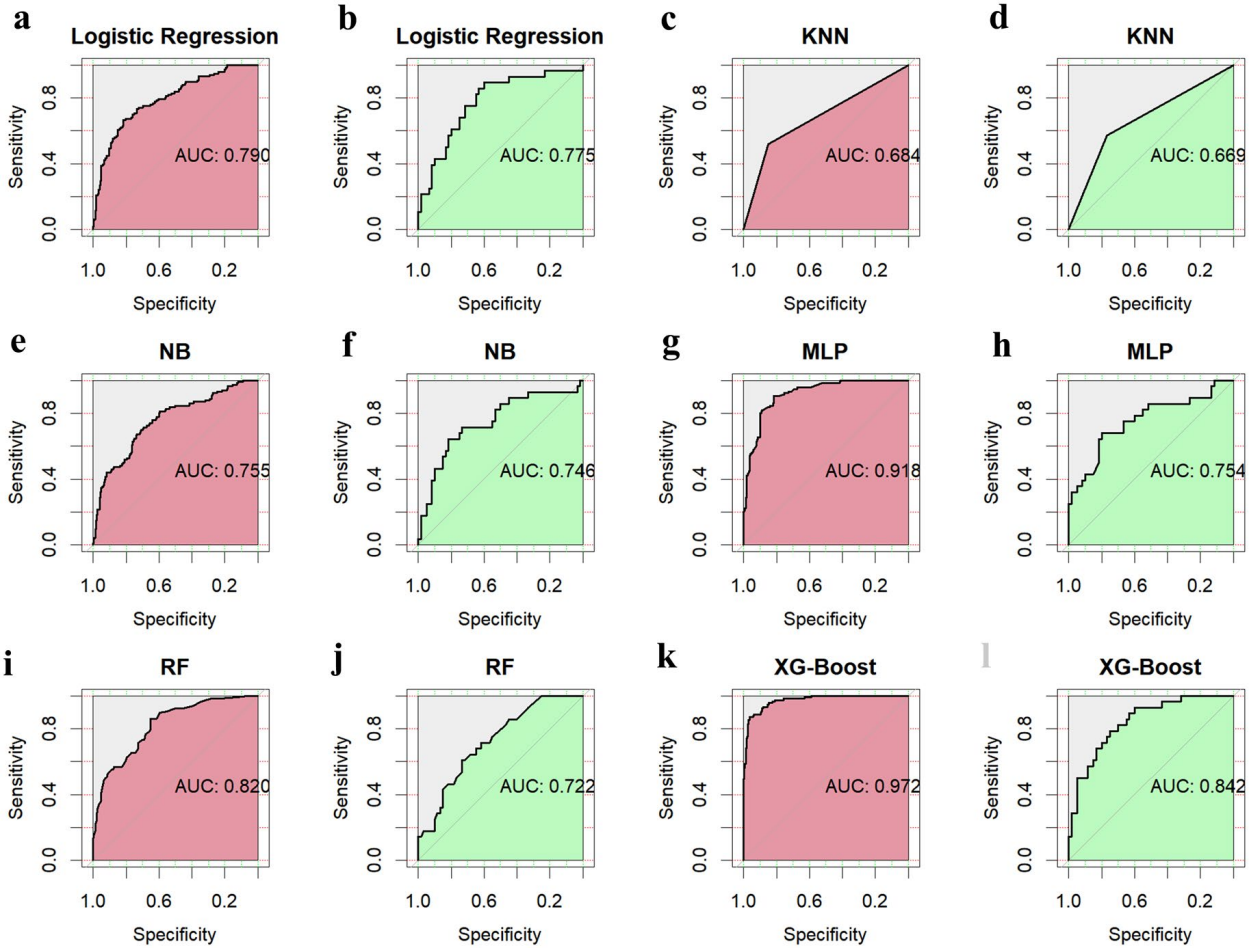
ROC Curves of Six Machine Learning Models, Red Represents the Training Set, Green Represents the Validation Set.

**Figure 5.**
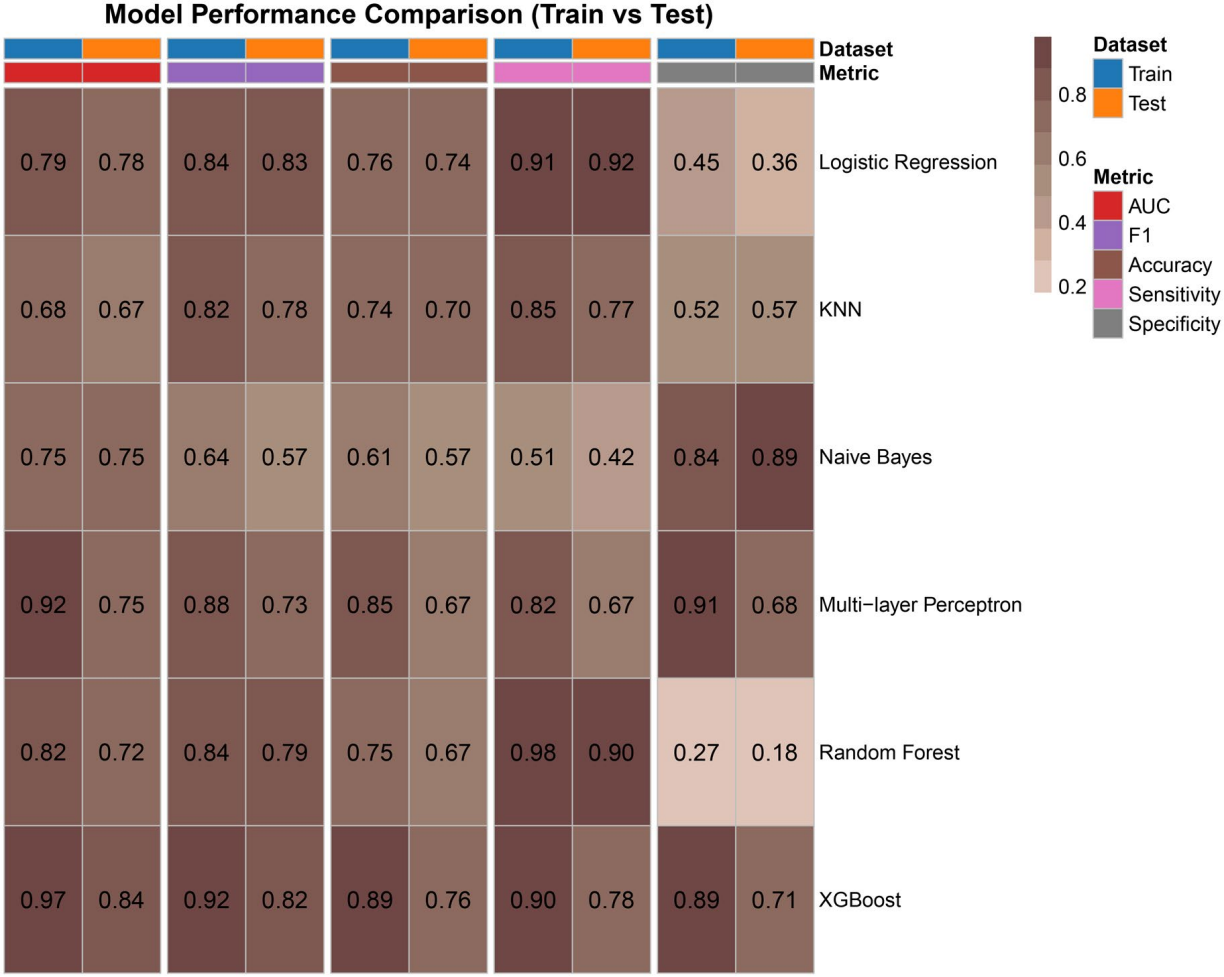
Comparison of Performance Among Different Machine Learning Models in the Training and Testing Sets. Each Cell Represents the Model’s Score on a Specific Dataset and Metric.

**Figure 6.**
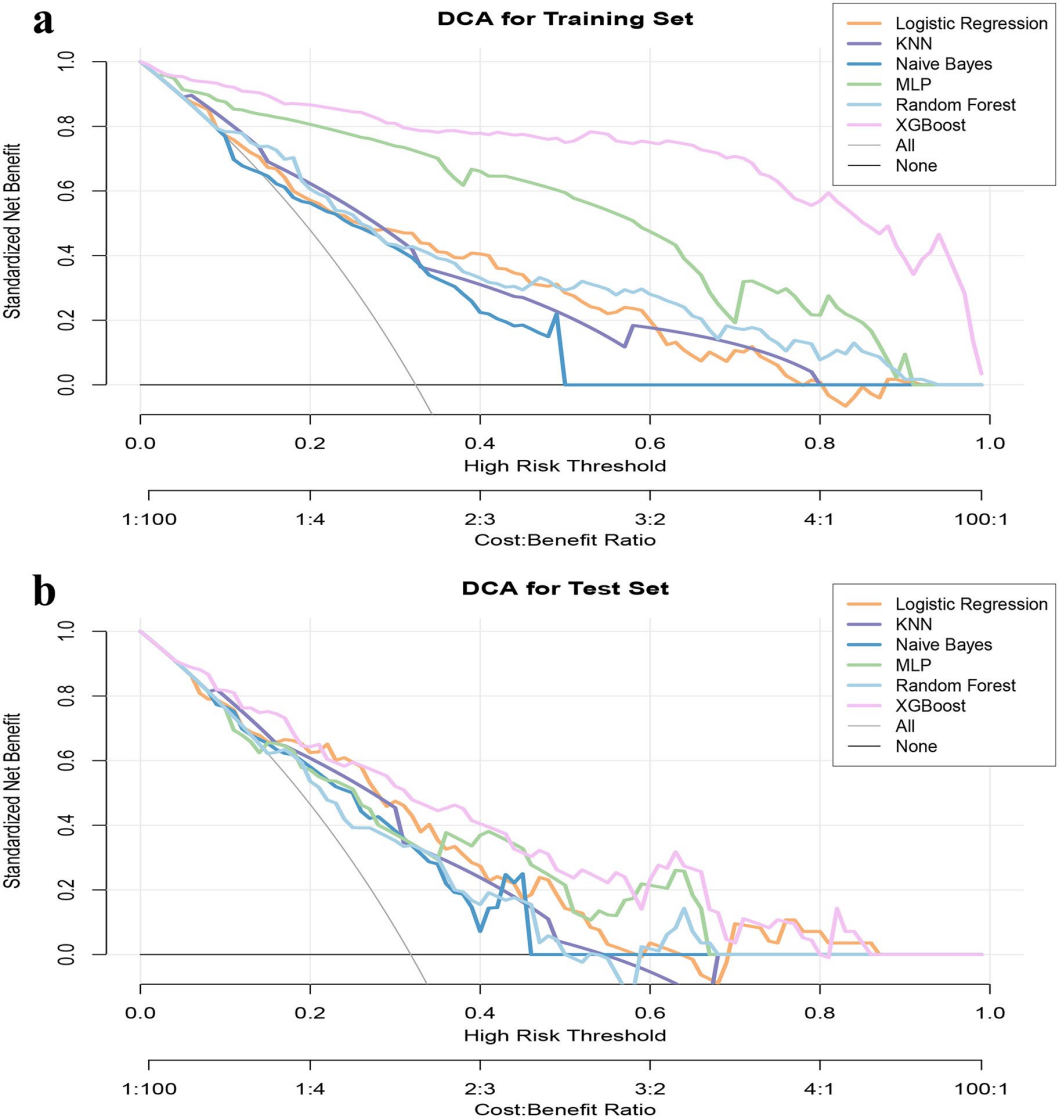
Decision Curve Analysis of Six Machine Learning Models, **(a)** Represents the Training Set, **(b)** Represents the Validation Set.

Among these models, XGBoost demonstrated superior predictive performance, achieving an AUC of 0.972 (95% CI: 0.958–0.987), with a sensitivity of 0.892, specificity of 0.905, accuracy of 0.897, and an F1-score of 0.921 **(**Figure **4****, Figure 5)**. In addition, the model yielded a positive predictive value (PPV) of 0.667, a negative predictive value (NPV) of 0.764, a positive likelihood ratio (PLR) of 4.186, and a negative likelihood ratio (NLR) of 0.646, further supporting its discriminative ability. To further validate the model’s generalizability, we evaluated its performance on an internal validation cohort (comprising 30% of the total samples). The XGBoost model maintained robust predictive ability in the validation set, yielding an AUC of 0.842 (95% CI: 0.757–0.926). To ensure the model’s reliability for clinical use, we performed a calibration analysis using the Hosmer-Lemeshow test. The results showed good calibration with χ^2^ = 7.352, *p* = 0.324 in the training set and χ^2^ = 9.538, *p* = 0.299 in the validation set, indicating that the predicted probabilities were well-aligned with the observed outcomes. To assess clinical applicability, we performed DCA for the XGBoost model. The results demonstrated favorable clinical utility across both the training set (Figure 6A) and the internal validation set (Figure 6B), supporting its potential for clinical decision-making in FS associated with EV infection.

### 3. 4.SHAP analysis and interpretation

**Figure 7.**
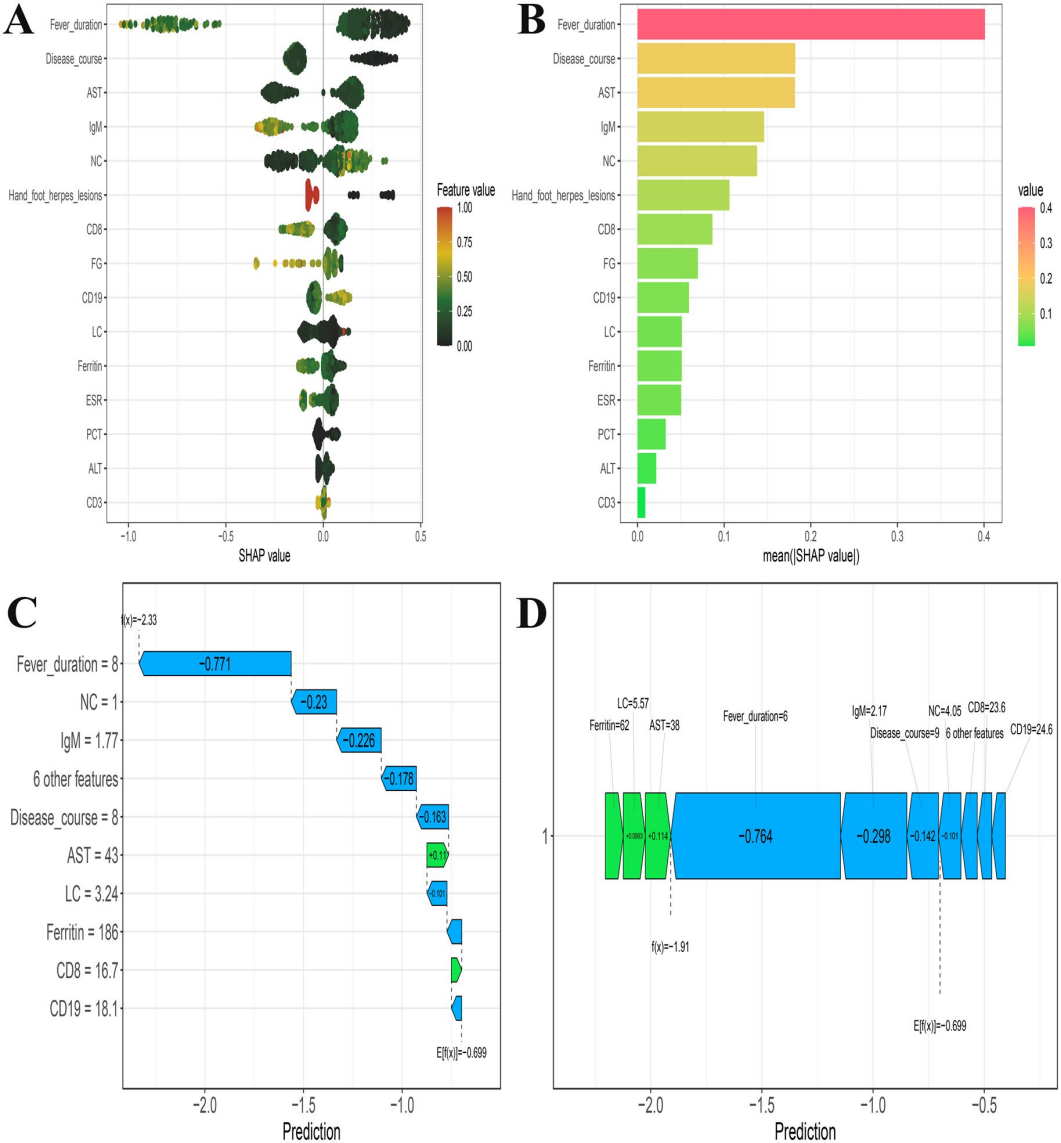
SHAP-based model interpretability visualizations for the XGBoost prediction model of febrile seizures. **(A)** Global SHAP summary plot showing the distribution of feature contributions across all patients; color indicates feature value (red = higher, green = lower). **(B)** Mean absolute SHAP values ranking the overall importance of features; fever duration and disease course are the strongest predictors. **(C)** Waterfall plot (Case 1) illustrating how individual features cumulatively increase or decrease the predicted risk of febrile seizures. **(D)** Waterfall plot (Case 2) demonstrating the patient-specific contributions of predictors to the final model output.

Enhancing the interpretability of the XGBoost model with SHAP, we generated a global summary plot to visualize the distribution of SHAP values for each selected variable across all patients (Figure 7A). We further ranked the mean absolute SHAP values to quantify the overall contribution of each predictor **(**Figure 7B). The most influential features were fever duration, disease course, AST, IgM, NC, hand–foot–mouth herpes lesions, CD8, FG, CD19, LC, ferritin, ESR, PCT, ALT, and CD3.

To demonstrate local interpretability, we randomly selected two representative patients. The SHAP waterfall plot for the first patient (Figure 7C) shows how individual features such as fever duration, NC, and IgM decreased or increased the predicted probability, cumulatively leading to the final model output. Similarly, for the second patient, the waterfall plot (Figure 7D) revealed opposite effects of predictors including fever duration, IgM, LC, and AST, thereby clarifying how the model generated individualized predictions.

**Figure 8.**
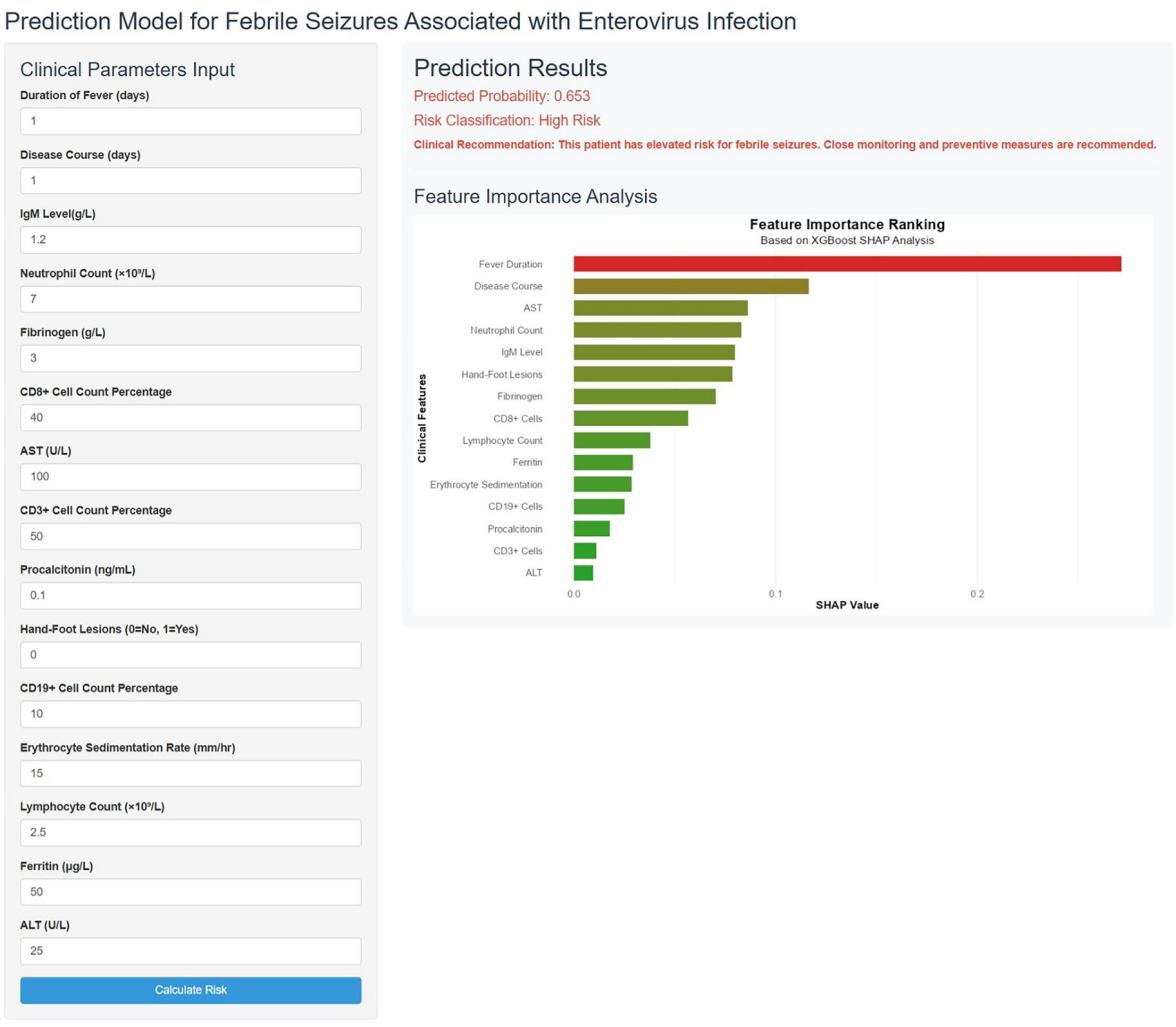
Shiny-based Web Calculator Model for Predicting Enterovirus-Induced Febrile Seizures. The Figure Displays the Input Clinical Parameters, Prediction Outcome (Risk Probability of 0.653), and SHAP-based Feature Importance Analysis, Ranking the Contribution of Each Clinical Feature to the Prediction Result.

To enhance visualization and facilitate clinical application, we developed a web-based calculator capable of estimating the probability of FS occurrence based on a child’s basic clinical information **(**Figure 8). For example, upon entering the following parameters for a child with EV infection: fever duration (1 day), disease course (1 day), IgM level (1.2 g/L), neutrophil count (7 × 10^9^/L), fibrinogen (3 g/L), CD8^+^ cell percentage (40%), AST (100 U/L), CD3^+^ percentage (50%), procalcitonin (0.1 ng/mL), absence of hand-foot lesions (0), CD19^+^ percentage (10%), ESR (15 mm/hr), lymphocyte count (2.5 × 10^9^/L), ferritin (50 µg/L), and ALT (25 U/L), the calculator yields a risk probability of 0.653. This classifies the patient as high risk and prompts a recommendation for close clinical monitoring. To facilitate access to the tool, the application can be accessed *via* the following link: https://lyh-123.shinyapps.io/shiny/

## 4. Discussion

 This study, based on ML techniques, developed and validated an interpretable ML model to predict FS in children after EV infection. Utilizing the XGBoost algorithm, the model incorporated 15 readily accessible clinical variables and demonstrated excellent predictive performance across both the training and internal validation cohorts. Notably, the study addressed the conventional “black-box” limitations of ML models by integrating SHAP for interpretability and deploying a web-based ‘Shiny’ calculator. This approach enabled early individualized FS risk quantification and stratification during the initial stages of hospitalization. Consequently, it provided clinicians with actionable insights for enhanced monitoring and potential early intervention in high-risk pediatric patients, thereby contributing to the reduction of unnecessary medical procedures and alleviating caregiver anxiety.

This study identified fever duration and length of hospital stay as key risk factors for predicting EV-associated FS. Notably, children in the FS group had significantly shorter fever duration and hospitalization than those without FS. Although fever is commonly associated with FS, there is no consistent temperature threshold known to trigger seizures[25]. Previous studies[26] have mainly emphasized the peak body temperature as a risk factor, while the rate of temperature rise has received limited attention.

However, the occurrence of FS is closely linked to age-dependent neuronal hyperexcitability triggered by fever, suggesting that excessive neuronal excitation plays a critical role in seizure onset[27,28]. In the context of EV infection, this study highlighted that the rate of fever escalation is a stronger predictor of FS rather than peak temperature or total duration of fever. This finding showed that, in specific viral contexts like EV infection, rapid temperature fluctuations may more readily induce seizures. The results suggest that EV may rapidly alter neuronal excitability thresholds, increasing FS risk even at relatively lower body temperatures in the early stages of illness. Clinically, this underscores the importance of vigilant monitoring for FS in children with EV infection, especially those presenting with a short duration of fever and rapid disease progression at admission.

This study also identified several biomarkers closely linked to inflammation and immune response as important predictors, including IgM levels, NC, PCT, FG, ferritin, ESR. These findings align strongly with the inflammatory hypothesis of FS[13,14,29]. EV infection triggers a strong systemic inflammatory response and leads to the release of numerous pro-inflammatory cytokines. These mediators not only cause fever but may also compromise the blood-brain barrier. They can activate glial cells in the central nervous system, disrupt neurotransmitter balance, and reduce the neuronal excitation threshold. Together, these effects increase the likelihood of seizures[8]. Although these inflammatory markers are not specific to EV infection, in our cohort they demonstrated predictive value for EV-associated FS because they reflect the intensity of systemic immune activation triggered by EV, rather than pathogen-specific mechanisms. In addition, while T-cell markers (CD3, CD4, CD8) were not found to be statistically significant in our cohort, the significant detection of the B-cell marker CD19 suggests that humoral immunity may play a more decisive role than cellular immunity in the pathogenesis of FS after EV infection. This novel finding highlights the need for further studies exploring both cellular and humoral immune mechanisms underlying FS in the context of EV infection. Nevertheless, we recognize that routinely testing immune markers is not cost-effective or sustainable in standard pediatric practice. While these parameters provide mechanistic insights into FS pathophysiology, simplified prediction models using readily available laboratory indicators may be more practical for real-world clinical application.

A key breakthrough of this study lies in overcoming a major barrier in the clinical application of ML models: the challenge of interpretability. By introducing SHAP value analysis, the study quantified the contribution of each predictive variable, clearly illustrating how various clinical features jointly influence the probability of FS. This enhanced both the transparency of the model’s predictions and clinicians’ trust and understanding of its reliability. Furthermore, the study developed and deployed an interactive web-based calculator using the Shiny framework. This tool transforms the complex XGBoost algorithm into an intuitive, user-friendly clinical interface. With just 15 routinely available clinical inputs, physicians can instantly obtain an individual child’s FS risk score and classification. The tool represents a shift from a “black-box” model toward an interpretable, actionable decision-support system for clinical use.

This study still has several limitations. First, it was conducted using data from a single center of Kunming Children’s Hospital, which may introduce selection bias. Therefore, the model’s external validity and generalizability require further confirmation. Second, the data used were primarily retrospective, carrying risks of missing values or inherent bias. Future prospective studies are needed to further validate the model’s accuracy and reliability. In addition, the model relies mainly on indicators available at admission or during the early phase of hospitalization. It does not account for the dynamic changes in laboratory markers and clinical symptoms as the disease progresses. Another important limitation is that FS were analyzed as a single group, without distinguishing between simple and complex FS. Since complex FS carries a higher clinical risk and may be a key factor in ICU admission, the lack of such data limits the interpretation of our findings. Moreover, we acknowledge that comparing XGBoost with more advanced gradient boosting algorithms, such as LightGBM and CatBoost, would provide deeper insights. In this study, we prioritized XGBoost due to its extensive application and strong track record in clinical prediction tasks. Future research should compare XGBoost with these alternative models to further validate its performance. Lastly, the model lacks a mechanism for continuous updating, which remains a critical direction for future research.

## 5. Conclusion

The XGBoost predictive model developed in this study demonstrated both high accuracy and clinical interpretability. The associated web-based calculator offers a new tool for risk stratification and management of FS in children with EV infections.

## Data Availability

The datasets generated and/or analyzed during the current study are not publicly available due to our research center policy, but are available from the corresponding author on reason-able request.
